# Glucose driven bacterial persistence in extensively drug-resistant tuberculosis with diabetes

**DOI:** 10.3389/fpubh.2026.1746879

**Published:** 2026-03-26

**Authors:** Xin Yao, Yarong Shi, Minghao Kong, Yujie Zhang, Jianxia Chen, Wenjuan Wang, Zhonghua Liu

**Affiliations:** 1 The Key Laboratory of Environmental Pollution Monitoring and Disease Control, Ministry of Education, School of Public Health, Guizhou Medical University, Guiyang, China; 2 Shanghai Key Laboratory of Tuberculosis, Shanghai Pulmonary Hospital, Tongji University School of Medicine, Shanghai, China

**Keywords:** bacterial clearance, diabetes mellitus, extensively drug-resistant tuberculosis, glucose, high bacterial load

## Abstract

**Background:**

Diabetes mellitus (DM) disrupts the metabolic environment of extensively drug-resistant tuberculosis (XDR) patients, promoting mycobacterial persistence. Key factors influencing bacterial clearance remain unclear. This study aimed to identify critical host factors and assess the therapeutic value of intensive glucose control.

**Method:**

A 48-month single-center retrospective cohort study compared sputum smear conversion (bacterial clearance) between 57 XDR-only (XDR) and 84 XDR with DM (XDR + DM) patients (similar treatment, consistent *in vitro* MIC resistance profiles). Kaplan–Meier analysis, Cox regression, generalized linear models, and mediation analysis were used to explore associations between diabetic status, blood glucose (GLU) levels, and bacterial clearance, as well as the impact of glucose control on treatment progression.

**Results:**

Compared with XDR patients, the XDR + DM group exhibited a significantly lower bacterial clearance rate (80.7% vs. 45.2%) and longer median clearance time (6 vs. 12 months), despite comparable baseline smear grades. Crucially, XDR + DM patients displayed distinct immunometabolic dysregulation, characterized by elevated inflammatory markers (CRP) and a disrupted CD4+ /CD8+ T-cell balance (increased CD4+, decreased CD8+ counts). Multivariable analysis identified hyperglycemia as the primary driver of delayed clearance (aHR = 1.48, 95% CI: 1.23–1.77, *p* < 0.001), serving as a significant mediator between DM and impaired outcomes (mediation effect: -0.82, *p* = 0.006). A glucose “dose–response” relationship was observed: severe hyperglycemia (GLU ≥ 11.1 mmol/L) markedly increased clearance delay risk (aHR = 5.29, *p* < 0.001), whereas optimal control (GLU < 7 mmol/L) mitigated these disadvantages.

**Conclusion:**

Dysregulated glucose metabolism and subsequent immune imbalance in diabetes are key drivers of delayed bacterial clearance in XDR patients, independent of the anti-tuberculosis regimen. Precise glucose control should be regarded as a core strategy, on a par with anti-XDR treatment.

## 1 Introduction

The therapeutic impasse in extensively drug-resistant tuberculosis (XDR) and its highly prevalent comorbidity, diabetes mellitus (DM), is a major challenge to global tuberculosis (TB) control (1). XDR is plagued by limited effective drugs, prolonged treatment, significant toxicities, and high costs, leading to persistently low standardized treatment coverage and hindering the 2035 TB elimination goal (2, 3). Even with novel drugs, treatment success remains suboptimal in refractory XDR cases, highlighting unresolved issues of inadequate efficacy and emerging resistance (4–6). Notably, DM and TB have a well-established bidirectional relationship (1): DM increases XDR risk 2- to 4-fold (7), while active TB impairs glucose control (1). Comorbid patients often present with higher sputum bacterial load and cavitary lesions, both linked to poorer prognosis (8–10), making this population a critical focus for clinical research.

Sputum smear conversion (SSC) is a key indicator of early treatment response and bacterial clearance in anti-TB therapy (11, 12), correlating with reduced infectivity, long-term success, and relapse risk (13, 14). Studies have found that delayed SSC can increase the risk of relapse by 2.3-fold (12). In XDR patients, limited bactericidal drugs prolong median SSC to 6–8 months (vs. 2–3 months in drug-sensitive TB) (12, 14). However, the biological drivers exacerbating this delay in diabetic hosts warrant deeper scrutiny. Recent mechanistic insights reveal that DM-associated metabolic dysregulation (specifically hyperglycemia and insulin resistance) disrupts the AMPK-mTOR signaling axis, thereby inhibiting the initiation of autophagy (15). This suppression critically impairs xenophagy, the selective autophagic process essential for sequestering intracellular *Mycobacterium tuberculosis* (*Mtb*) (16, 17). Furthermore, the compromised autophagic flux in DM impedes phagosome-lysosome fusion, preventing the effective lysosomal degradation of the pathogen (17). Consequently, this failure of innate immunity allows *Mtb* to establish a persistent intracellular niche and evade clearance (18). While these molecular defects offer a compelling explanation for bacterial persistence, the extent to which these metabolic-immune interactions quantitatively translate to delayed SSC in the clinical XDR population remains insufficiently explored.

Given the multidrug-resistant nature of XDR and limitations of clinical treatments, XDR with XDR (DM+XDR) patients have a significantly lower cure rate. Whether non-pharmacological interventions can enhance XDR bacterial clearance in this population urgently requires investigation. Based on consistent treatment strategies and uniform *in vitro* MIC resistance profiles across all participants, this single-center retrospective study enrolled 57 XDR and 84 DM+XDR patients. Using multidimensional statistical approaches, it aims to clarify DM’s specific impact on XDR bacterial clearance and identify key non-pharmacological regulatory factors. Addressing the research gap in impaired clearance among DM+XDR patients, this study is expected to support optimizing clinical strategies, enhancing clearance via intensive glucose management, improving outcomes, and reducing the overall disease burden in tuberculosis-diabetes comorbid populations.

## 2 Method

### 2.1 Ethics statement

Our experiments were in accordance with the ethical standards formulated in the Helsinki Declaration. This study was approved by the Ethics Committee of Shanghai Pulmonary Hospital, Tongji University School of Medicine (Shanghai, China) (K19-060Y). All participants gave verbal consent for the use of their clinical information for research purposes.

### 2.2 Study patients

**Figure 1:**
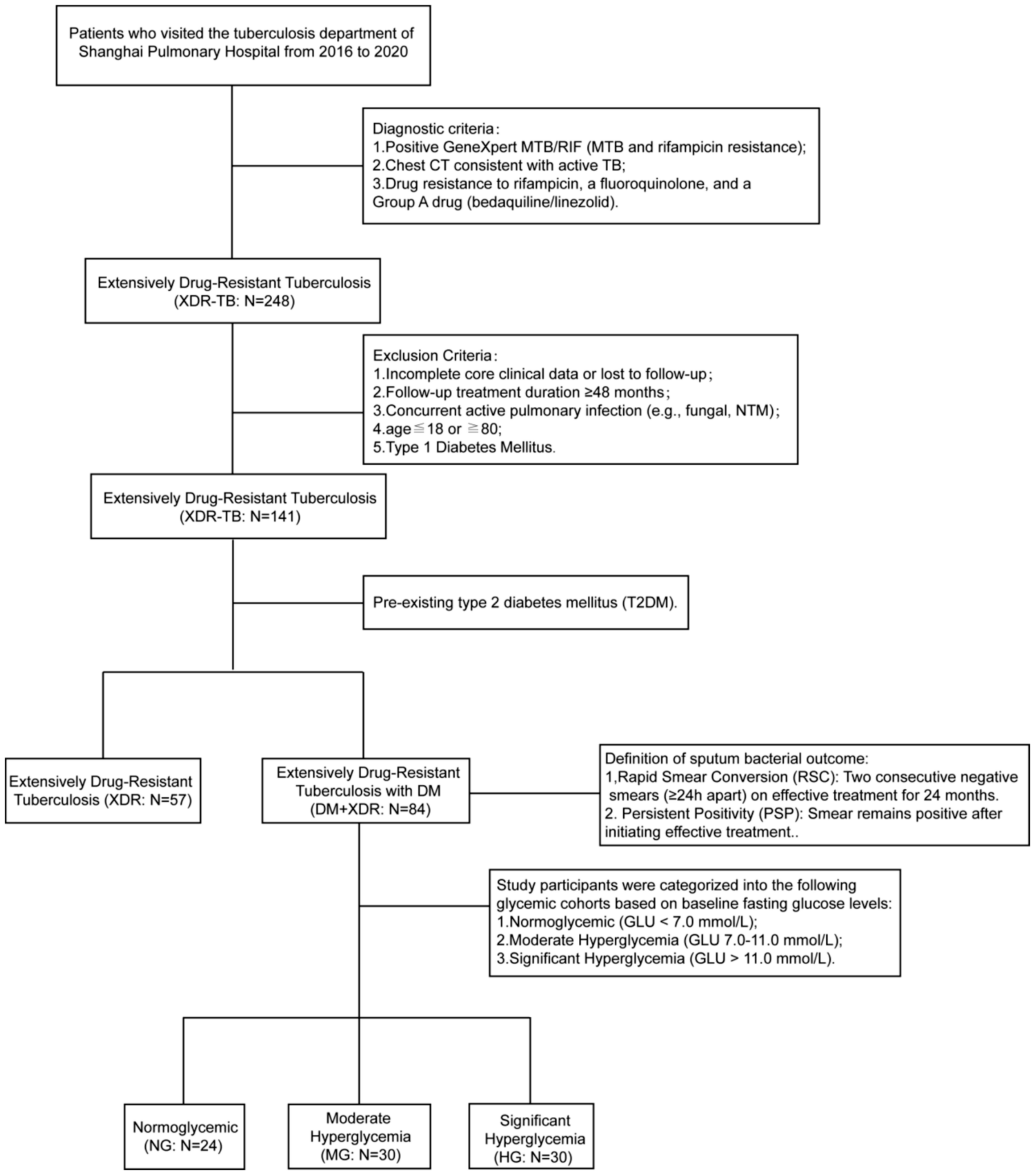
Patient enrolment and outcomes.

This single-centre, retrospective cohort study analysed patients with XDR admitted to Shanghai Pulmonary Hospital between 2016 and 2020. We consecutively enrolled all adult patients (aged >18 years) with microbiologically confirmed XDR for a 48-month analysis. Based on clinical history, patients were stratified into two groups: those with XDR alone (XDR) and those with XDR and comorbid type 2 diabetes (DM+XDR). Exclusion criteria included: patients with numerous comorbidities or with type 1 diabetes, those with incomplete medical records, those who transferred to another hospital or died during treatment, and those who declined to participate in the study (see Figure 1). Ultimately, 141 patients were included in the final analysis (XDR, *n* = 57; DM+XDR, *n* = 84).

### 2.3 Baseline data collection

Data on baseline characteristics and clinical information were collected from the electronic medical record system of Shanghai Pulmonary Hospital and compiled into a database, including: (a) Demographic and epidemiological data: age, sex, height, weight, clinical symptoms (e.g., fever, cough), and medical history; (b) Clinical disease indicators: sputum smear positivity grade (assessed using the internationally standardized 1–4 + grading system), and chest CT imaging findings (e.g., cavity formation, pleural effusion, and involved lung regions); (c) Treatment regimen and drug resistance profile: detailed records of anti-tuberculosis medications and drug susceptibility testing results; (d) Biochemical parameters: a total of 31 indicators across four categories—infection and inflammation markers (e.g., WBC, NEUT, CRP), immune markers (e.g., PLT, CD4, CD8), metabolic markers (e.g., CYSC, GLU), and nutritional markers (e.g., ALB, PA).

### 2.4 Definition

#### 2.4.1 Primary outcomes

Sputum smear conversion (SSC) and time to sputum smear conversion (TSSC) were defined as the interval from the initiation of anti-tuberculosis treatment until the first instance of two consecutive negative sputum smears for acid-fast bacilli (collected at least 1 month apart) (19). Patients who did not meet this criterion by the end of the planned treatment course were classified as non-converters, with the treatment completion date recorded as the censoring time. Based on conversion within 24 months under an effective regimen, patients were categorized as having rapid sputum conversion (RSC) or persistent smear positivity (PSP). Smear grading was performed by examining stained slides under microscopy for acid-fast bacilli (AFB). Smears were classified as: (1) Negative: no AFB observed in 100 fields; (2) Positive (graded 1 + to 4+): 1 + (1–9 AFB per 100 fields); 2 + (1–9 AFB per 10 fields); 3 + (1–9 AFB per field); 4 + (≥10 AFB per field).

#### 2.4.2 Secondary outcome

Treatment outcomes were defined according to the WHO definitions and reporting framework for tuberculosis (19). Clinical cure was defined as completion of treatment with at least two subsequent negative bacteriological tests, symptomatic improvement, and ≥50% resolution of lesions on chest CT imaging. Treatment failure was defined as a positive mycobacteriological test or lack of clinical improvement.

### 2.5 Glucose grouping method

Subgroup analysis of the DM+XDR group: To elucidate the dose–response relationship between glucose levels and treatment outcomes, patients in the DM+XDR group were stratified into three subgroups based on baseline fasting plasma glucose (FPG) levels: ① normal glucose subgroup (GLU < 7 mmol/L, NG, *n* = 24); ② moderate hyperglycemia subgroup (7 mmol/L ≤ GLU < 11 mmol/L, MG, *n* = 30); and ③ severe hyperglycemia subgroup (GLU ≥ 11 mmol/L, HG, *n* = 30). The stratification criteria were established with reference to the glucose control targets and diagnostic criteria outlined in the Chinese Guidelines for the Prevention and Treatment of Type 2 Diabetes (2023 Edition). No statistically significant differences were observed across subgroups in terms of age, sex, or treatment regimen composition (all *p* > 0.05).

### 2.6 Statistical analysis

Statistical analyses were conducted using SPSS 26.0 and R 4.3.1, with figures generated in GraphPad Prism 9.5 and Origin 2024. Categorical demographic variables were compared using the *χ*^2^ test or Fisher’s exact test, and continuous variables were tested for normality with the Shapiro–Wilk test. Normally distributed data are expressed as mean ± SD and compared by independent samples *t*-test; non-normal data are summarized as median (IQR) and analyzed using the Mann–Whitney U test. Categorical variables are presented as *n* (%). Kaplan–Meier analysis was used to evaluate cumulative cure and bacterial clearance rates. A generalized linear model (GLM) was applied to identify factors associated with sputum smear conversion, reporting standardized coefficients (*β*). Mediation analysis was performed using the Hayes PROCESS macro, with bootstrap sampling (5,000 iterations) to estimate 95% CIs; effects were deemed significant if the CI excluded zero. Within the DM+XDR subgroup, a Cox proportional hazards model assessed the effect of glucose levels on bacterial clearance, providing HRs and 95% CIs. All tests were two-sided, with *p* < 0.05 considered statistically significant.

## 3 Results

### 3.1 DM exacerbates disease severity and impairs treatment response in XDR

Existing evidence confirms that extensively drug-resistant tuberculosis (XDR) comorbid with diabetes mellitus (DM; DM+XDR) is associated with poor clinical outcomes, including high bacillary load, cavitary lesions, and treatment failure (8, 10). Compared to patients with XDR alone, those with comorbid DM (DM+XDR) were significantly older and predominantly male (Table 1). Despite comparable sputum smear grades at baseline, the DM+XDR group exhibited a higher proportion of patients with a high bacterial load (4+) (8.8% vs. 23.8%, *p* = 0.025). Radiologically, DM+XDR patients presented with more severe pulmonary pathology, characterized by increased frequencies of cavitary lesions and pleural effusions, as well as more extensive lung involvement. Treatment outcomes were markedly inferior in the comorbid group: the sputum culture conversion rate was significantly lower (45.2% vs. 80.7%), and the median time to conversion was prolonged (12 months vs. 6 months).

**Table 1:** Baseline clinical characteristics of patients with XDR-TB alone (XDR) and those with XDR-TB and diabetes (DM+XDR) (*N* = 141).

Variable	Total (*n* = 141)		XDR (*n* = 57)		DM+XDR (*n* = 84)		*p*-value
Age	47.84 ± 13.60		39.95 ± 13.72		53.20 ± 10.64		<0.001
Sex							<0.001
Female	34	24.11	26	45.61	8	9.52	
Male	105	74.47	31	54.39	76	90.48	
BMI	21.79 ± 2.93		21.46 ± 2.10		22.02 ± 3.80		0.271
Sputum smear							0.057
1+	36	25.53	18	31.58	18	21.43	
2+	36	25.53	18	31.58	18	21.43	
3+	44	31.21	16	28.07	28	33.33	
4+	25	17.73	5	8.77	20	23.81	
Sputum smear turns negative	84	59.57	46	80.70	38	45.24	<0.001
Median time for sputum smear to turn negative	8(3,14)		6(2,7)		12(4,18)		<0.001
CT features of lungs							
Cavity	109	77.30	38	66.67	71	84.52	0.015
Pleural effusion	40	28.37	6	10.53	34	40.48	<0.001
The affected area of the lesion							0.002
Left/right	33	23.40	21	36.84	12	14.29	
Both	108	76.60	36	63.16	72	85.71	
Tuberculosis treatment history	141	100.00	57	100.00	84	100.00	1.000
Adverse drug reactions	64	45.39	25	43.86	38	45.24	1.000
Cure	74	52.48	39	68.42	35	41.67	0.002

**Figure 2:**
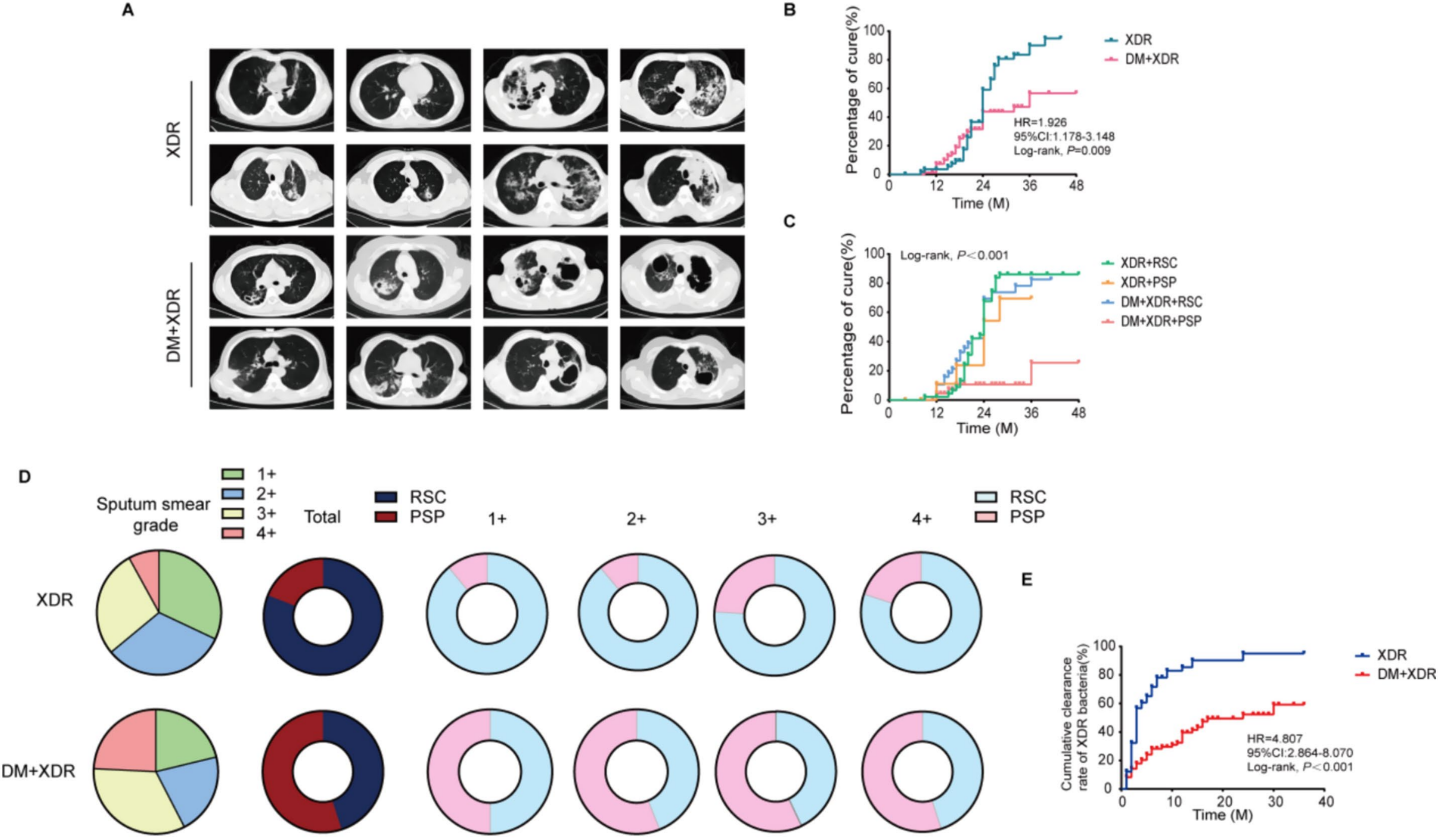
Clinical impact of diabetes on disease severity and treatment response. **(A)** Representative chest CT images from a patient with XDR alone (upper panel) and a patient with XDR and diabetes (DM+XDR, lower panel). **(B)** Kaplan–Meier curves comparing cumulative treatment success rates between patients with XDR alone and those with XDR-DM (log-rank test). **(C)** Cumulative treatment success rates, stratified by bacteriological outcomes, comparing the two groups (Kaplan–Meier analysis). Treatment success is defined as a composite endpoint combining cure and treatment completion without evidence of failure. **(D)** Stacked bar charts showing the distribution of patients by baseline sputum smear grade (1+, 2+, 3+) at treatment initiation (left), and the corresponding rates of bacteriological clearance achieved by the end of intensive phase (right), presented separately for each group. **(E)** Kaplan–Meier curves comparing cumulative bacterial clearance rates between the two groups (log-rank test). RSC, Sustainable bacterial clearance; PSP, Persistent bacterial positivity throughout the planned treatment duration.

Survival analysis further confirmed the divergent outcomes (Figure 2). The cumulative treatment success rate was significantly lower in the DM+XDR group compared to the XDR group (Log-rank *p* = 0.009; Figure 2B). Pulmonary CT imaging quantified larger cavity volumes in comorbid patients (Figure 2A). Longitudinally, DM+XDR patients maintained higher bacterial loads throughout the treatment course, with lower conversion rates observed across all baseline smear grades (Figure 2D). A strong correlation was identified between early bacterial clearance and final treatment success (Log-rank *p* < 0.001; Figure 2C). Dynamic analysis demonstrated that the cumulative probability of bacterial clearance remained persistently lower in the DM+XDR group throughout the observation period (Log-rank *p* < 0.001; Figure 2E). These findings indicate that DM is associated with delayed bacterial clearance and treatment failure, independent of the initial drug resistance profile (Table 2, *p* > 0.05).

**Table 2:** Comparison of treatment regimens and drug resistance profiles between groups.

Variable	XDR (*n* = 57)		DM+XDR (*n* = 84)		*p*-value
Use second-line drugs in the treatment plan					
Bedaquiline	10	17.54	18	21.43	0.669
Linezolid	30	52.63	44	52.38	1.000
Clofazimine	38	66.67	53	63.10	0.722
Cycloserine	44	77.19	67	79.76	0.834
Drug resistance spectrum					
Number of first-line drug resistance types (Median [IQR])	4 (3, 5)		4 (3, 5)		0.882
Number of second-line drug resistance types (Median [IQR])	5 (4, 7)		6 (5, 7)		0.574
Resistance to specific drugs					
Fluoroquinolones	57	100.00	84	100.00	1.000
Second line injection	42	73.68	62	73.81	1.000
Bedaquiline	15	26.32	24	28.57	0.849
Linezolid	12	28.07	31	32.14	0.789

### 3.2 Hyperglycemia mediates the impaired bacterial clearance in DM+XDR

**Figure 3:**
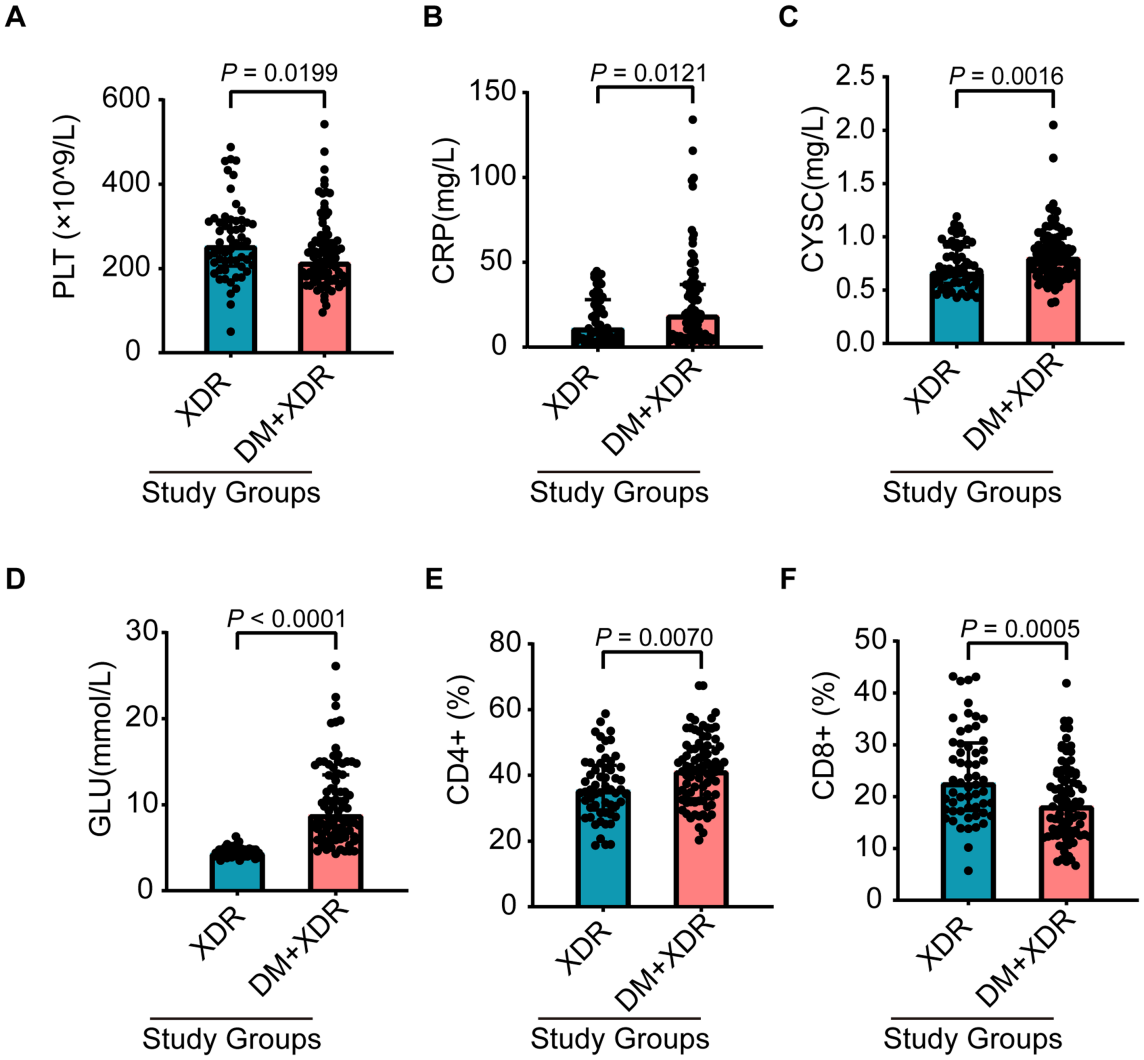
Comparison of laboratory and immunological parameters between XDR and DM+XDR. **(A–F)** Scatter plots comparing the levels of six key biomarkers—PLT **(A)**, CRP **(B)**, CYSC **(C)**, GLU **(D)**, CD4 **(E)**, and CD8 **(F)**—between the two groups. Between-group differences were assessed using the Mann–Whitney *U* test, with a *p* value < 0.05 considered statistically significant.

**Figure 4:**
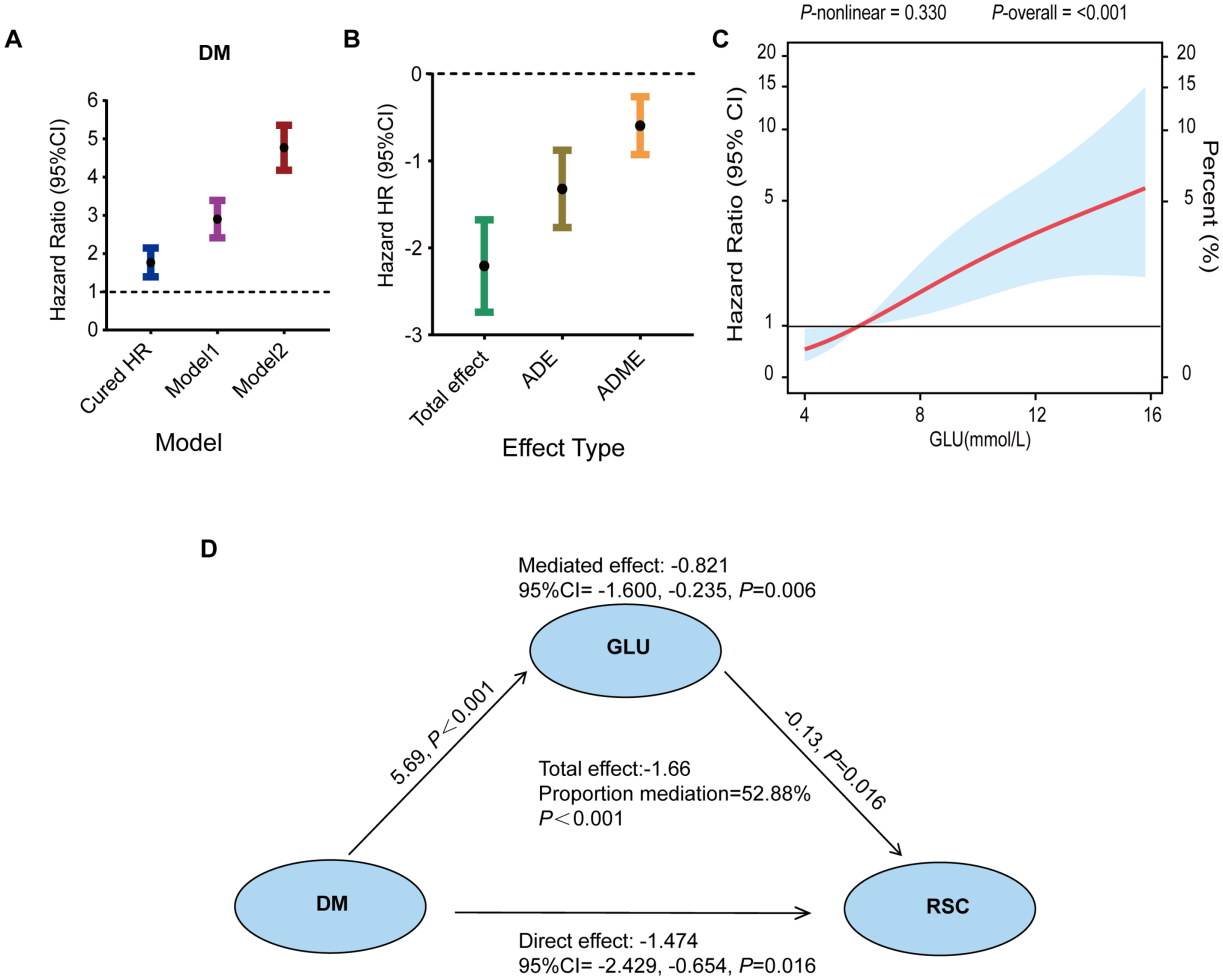
Hyperglycaemia mediates the negative effect of diabetes on bacterial clearance. **(A)** Progressive adjustment in Cox models reveals the persistent association between diabetes and delayed bacterial clearance after accounting for clinical confounders. **(B)** Mediation analysis using the bootstrap method confirms that a significant portion of diabetes’ total effect is indirectly mediated through elevated GLU levels (ACME), alongside a residual direct effect (ADE). **(C)** The continuous relationship between rising GLU levels and increasing risk of delayed clearance is visualized using a restricted cubic spline model. **(D)** Schematic of the established mediation pathway, quantifying the significant indirect effect of diabetes via GLU, as tested by bootstrap. All associations were significant at *p* < 0.05.

Despite receiving similar treatment regimens (Table 2, *p* > 0.05), outcomes differed significantly between groups. To identify host factors contributing to this discrepancy, we compared 31 biochemical and immunological parameters (Supplementary Table S1). The DM+XDR group exhibited a distinct systemic profile characterized by significantly reduced platelet (PLT) and CD8^+^ T-cell counts, alongside markedly elevated levels of C-reactive protein (CRP), cystatin C (CYSC), blood glucose (GLU), and CD4^+^ T-cell counts (Figures 3A–F). To determine independent predictors of delayed bacterial clearance, the six identified differential markers were entered into a generalized linear model (Table 3). In univariable analysis, only GLU (HR = 1.586, 95% CI: 1.326–1.898) and CRP (HR = 1.031, 95% CI: 1.006–1.057) were significant risk factors. After adjusting for age, sex, and baseline lung injury, both GLU (adjusted HR = 1.477, 95% CI: 1.229–1.774) and CRP (adjusted HR = 1.038, 95% CI: 1.013–1.065) remained independently associated with delayed clearance. Mediation analysis was performed to disentangle the causal pathways (Figure 4). A significant indirect effect was observed only for GLU (effect size = −0.821, 95% CI: −1.600 to −0.235, *p* = 0.006), while no significant mediation was found for CRP or other markers (Supplementary Figure S1). These results identify blood glucose levels as a key mediator linking DM to impaired bacterial clearance in XDR.

**Table 3:** Generalized linear model analysis of serum biomarkers associated with time to bacterial clearance.

Indicator variable	Cured HR (95% CI)	*p*-value	Model 1	*p*-value	Model 2	*p*-value
			HR (95% CI)		HR (95% CI)	
PLT	1.011 (0.991, 1.032)	0.284	1.008 (0.988, 1.029)	0.426	1.006 (0.986, 1.027)	0.561
CYSC	1.328 (0.847, 2.079)	0.216	0.675 (0.430, 1.060)	0.088	1.520 (0.964, 2.392)	0.072
GLU	1.586 (1.326, 1.898)	<0.001	1.521 (1.270, 1.821)	<0.001	1.477 (1.229, 1.774)	<0.001
CD4+	1.070 (0.989, 1.157)	0.091	1.062 (0.982, 1.148)	0.128	1.053 (0.973, 1.139)	0.206
CD8+	1.018 (0.935, 1.109)	0.684	1.004 (0.922, 1.094)	0.921	1.005 (0.921, 1.096)	0.913
CRP	1.031 (1.006, 1.057)	0.016	1.036 (1.011, 1.063)	0.005	1.038 (1.013, 1.065)	0.003

Crude HR represents the unadjusted association between each factor and the outcome variable, without controlling for any confounding factors.

Model 1: Adjusted for demographic characteristics, including sex, age.

Model 2: Adjusted for factors in Model 1 plus cavity, pleural effusion and the affected area of the lesion.

### 3.3 Dose–response relationship between blood glucose and treatment outcomes

**Figure 5:**
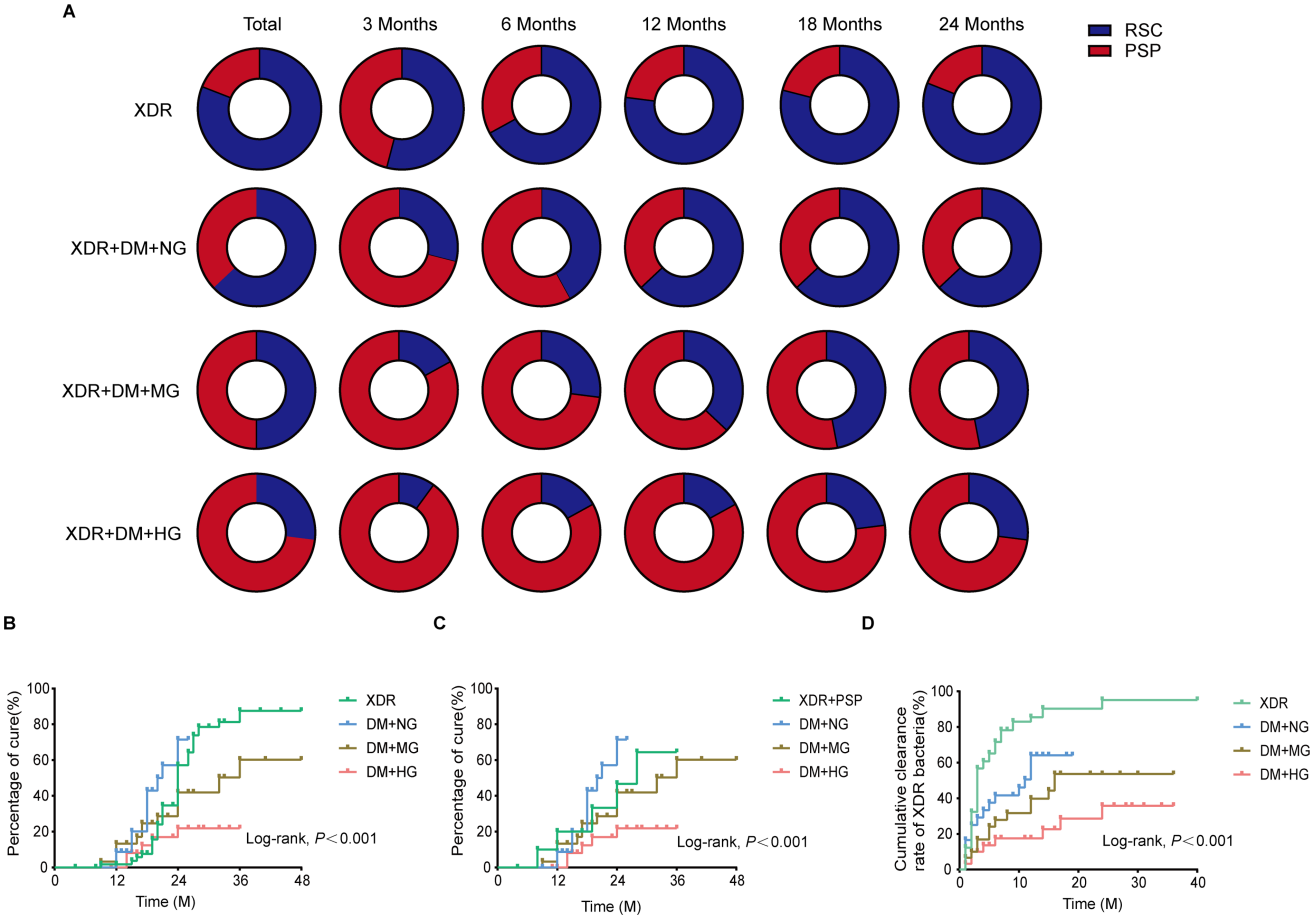
Gradient effect of blood glucose levels on bacterial clearance and treatment success. **(A)** Proportional rates of bacterial clearance at months 3, 6, 12, 18, and 24 of treatment, stratified by baseline blood glucose subgroups. **(B)** Cumulative treatment success rates for patients with XDR alone and for the different blood glucose subgroups (Kaplan–Meier analysis). **(C)** Cumulative treatment success rates, with the XDR alone group partitioned into those achieving sustained bacterial clearance (RSC) versus others, compared against the different blood glucose subgroups (Kaplan–Meier analysis). **(D)** Cumulative bacterial clearance rates across the different blood glucose subgroups (Kaplan–Meier analysis).

To assess the impact of glucose (GLU) control, DM+XDR patients were stratified into normoglycemia (NG: GLU < 7 mmol/L), moderate hyperglycemia (MG: 7 ≤ GLU < 11.1 mmol/L), and severe hyperglycemia (HG: GLU ≥ 11.1 mmol/L) subgroups (Supplementary Table S2). A clear dose–response relationship was observed: higher glucose levels correlated with progressively delayed bacterial clearance and poorer treatment outcomes (Figure 5). The cumulative cure rate in the HG subgroup was only 20.0%, significantly lower than in the NG and MG subgroups. The median time to bacterial clearance was prolonged to 12 months in the MG group and 14 months in the HG group, compared to 6 months in the NG group (Figure 5A). Survival analysis showed significant differences in both cumulative clearance and treatment success across glucose strata (Log-rank *p* < 0.001; Figures 5B,D). Notably, the treatment success rate in the NG subgroup was comparable to that of the non-diabetic XDR group (Figure 5C). In the multivariable Cox proportional hazards model (Table 4), the risk of delayed bacterial clearance increased with the severity of hyperglycemia. Compared to the XDR group, the adjusted hazard ratios for delayed clearance were 2.29 (95% CI, 1.14–4.61, *p* = 0.020) for the MG group and 5.29 (95% CI, 2.32–12.05, *p* < 0.001) for the HG group. However, the risk in the NG group was not statistically significant after adjustment (adjusted HR = 1.97, 95% CI: 0.98–3.96, *p* > 0.05), suggesting that strict glycemic control may mitigate the adverse effects of DM on XDR treatment response.

**Table 4:** Multivariable Cox proportional hazards analysis of blood glucose strata as a risk factor for bacterial persistence.

Grouping variable	Cured HR (95% CI)	*p*-value	Model 1		Model 2	
			HR (95% CI)	*p*-value	HR (95% CI)	*p*-value
XDR	Ref		Ref		Ref	
DM+NG	1.972 (1.091, 3.571)	0.025	1.555 (0.832, 2.907)	0.167	1.497 (0.776, 2.890)	0.228
DM+MG	2.506 (1.828, 6.135)	<0.001	2.463 (1.266, 4.785)	0.008	2.294 (1.140, 4.608)	0.020
DM+HG	6.849 (3.175, 14.925)	<0.001	5.587 (2.513, 12.500)	<0.001	5.291 (2.320, 12.048)	<0.001

Crude HR represents the unadjusted association between each factor and the outcome variable, without controlling for any confounding factors.

Model 1: Adjusted for demographic characteristics, including sex, age.

Model 2: Adjusted for factors in Model 1 plus cavity, pleural effusion and the affected area of the lesion.

## 4 Discussion

The co-occurrence of extensively drug-resistant tuberculosis (XDR) and diabetes mellitus (DM) has emerged as a major challenge in tuberculosis control (1). This study reveals that the clinical cure rate among patients with this comorbidity was only 41.67%, more than a quarter lower than that in the XDR (68.42%), underscoring the urgency and complexity of their clinical management. Critically, DM patients exhibit persistently high pulmonary bacterial loads, and when anti-tuberculosis drug efficacy is compromised by drug resistance, glucose control becomes the dominant factor determining treatment outcomes. DM is not merely a comorbidity but, through the central pathway of hyperglycemia, sustains a high bacterial burden, exacerbates pulmonary damage, impairs the host’s clearance capacity, prolongs the natural course of XDR, and ultimately leads to a clinical dilemma in which uniform therapeutic strategies yield divergent outcomes. This finding elucidates why some patients with XDR and DM maintain persistently high bacterial loads and experience poor prognosis, establishing precise glucose management as a key modifiable target to improve treatment response.

Clinical management of dR-TB has long centered on pathogen drug susceptibility profiles, with treatment regimens heavily relying on *in vitro* drug susceptibility testing results (20). However, this study demonstrates that DM is a key variable driving significant differences in clinical outcomes among XDR patients even with similar treatment backgrounds. Despite comparable baseline sputum smear positivity rates between groups, DM constitutes a structural barrier to sustaining high pulmonary bacterial loads, manifested by universally reduced clearance capacity across all sputum bacteriological grades. Persistent bacterial presence induces host matrix metalloproteinases, triggering active caseation and further exacerbating tissue damage (21–23). Comorbid patients exhibit more severe lung structural destruction, with imaging showing higher cavity formation and pleural effusion rates, wider lesion infiltration, and larger cavity volumes (24)—features closely linked to chronic hyperglycemia-induced pulmonary metabolic disturbances and local immune dysregulation (1), which synergistically worsen treatment outcomes (1, 25). Additionally, patients achieving sputum culture conversion during treatment have significantly better outcomes than those with persistent positivity, indicating that bacterial persistence is not only a marker of treatment failure but also a direct driver of lung tissue destruction, forming a vicious cycle of pathological progression and poor prognosis (26). This necessitates a shift in clinical management from a singular focus on “which drugs to use” to simultaneous attention to “which host is being treated” for more precise comorbidity management.

As a chronic inflammatory disease, DM directly or indirectly impairs the generation and function of T-helper (Th) cells (27). Focusing on the host milieu rather than pathogen genetics, this study reveals that DM profoundly remodels the overall internal environment of XDR patients, presenting distinct immunometabolic dysregulation phenotypes: persistent elevation of C-reactive protein (CRP) indicates occult systemic inflammation that impairs immune surveillance and accelerates lung tissue destruction (28, 29). Crucially, the observed aberrant CD4+/CD8 + T-cell ratio changes are driven by specific molecular pathways of glucotoxicity (27). Mechanistically, chronic hyperglycemia accelerates the formation of advanced glycation end-products (AGEs), which interact with their receptors (RAGE) on T-cells to trigger intracellular oxidative stress and mitochondrial dysfunction (21, 30). This signaling cascade preferentially induces apoptosis in CD4+ Th1 cells while impairing their proliferative capacity, thereby disrupting the adaptive immune architecture essential for granuloma maintenance and bacterial containment (31). Furthermore, decreased platelet counts with elevated cystatin C levels suggest insidious diabetic microangiopathy and early renal impairment risk (32, 33). These changes collectively reflect widespread multisystem damage from hyperglycemia-induced metabolic disturbances, indirectly compromising host reserve capacity against chronic infection (34). Further analysis identifies blood glucose (GLU) and CRP as the only key independent factors reducing bacterial clearance efficacy, with mediation analysis confirming GLU as the central bridge linking DM to impaired clearance (26, 27). Hyperglycemia provides a favorable metabolic niche for *Mtb*, while active tuberculosis exacerbates glucose metabolic disorders, forming a bidirectional vicious cycle (1, 26, 27). This study redefines DM from a vague comorbid diagnosis to a precise, quantifiable core pathological entity driven primarily by sustained Hyperglycemia, laying a critical theoretical foundation for targeted glucose intervention.

Hyperglycemia and *Mtb* infection form a bidirectional vicious cycle, mutually exacerbating each other and leading to difficulties in pathogen clearance and loss of metabolic homeostasis (27). Glycated hemoglobin (HbA1c), a core DM biomarker, predicts drug-resistant tuberculosis outcomes and sputum culture conversion probability (22, 35). Stratifying comorbid patients by glucose levels, this study demonstrates a progressive decline in bacterial clearance capacity and stepwise reduction in cumulative treatment success with increasing blood glucose. Notably, the severe hyperglycemia (HG) subgroup had a treatment success rate of only 20%, with a > 5-fold higher risk of delayed bacterial clearance than XDR alone after adjusting for confounders. This clinical observation is underpinned by distinct biological mechanisms. Beyond simply providing an enriched metabolic substrate for bacterial growth, uncontrolled hyperglycemia fundamentally undermines host innate immunity by suppressing autophagy (36–38). Recent mechanistic studies indicate that high glucose levels directly inhibit the autophagic flux (specifically xenophagy) required for the intracellular elimination of mycobacteria (36). Under hyperglycemia conditions, the expression of key autophagy-related proteins is downregulated, and the fusion of autophagosomes with lysosomes is impaired (39). This blockade prevents the effective degradation of *Mtb* within macrophages, allowing the pathogen to evade immune surveillance and establish a persistent intracellular reservoir even under antibiotic pressure (18, 36). Consequently, the inability to clear intracellular bacteria due to autophagy suppression likely drives the persistently high bacterial loads and delayed sputum conversion observed in our uncontrolled DM cohort. Conversely, comorbid patients with optimal glucose control (NG subgroup) achieved a cumulative cure rate comparable to XDR and superior to XDR patients with failed bacterial clearance, indicating that strict glucose control can restore these innate defense mechanisms and almost fully offset DM-related therapeutic disadvantages (40). Despite challenges from overlapping toxicities between hypoglycemia and anti-tuberculosis drugs (41), active and strict glucose control should be elevated to a core priority equivalent to anti-tuberculosis therapy in the overall XDR treatment framework. This study provides high-quality evidence-based support for the comprehensive integration of systematic glucose monitoring and management into drug-resistant tuberculosis guidelines.

This study has several limitations. As a cross-sectional investigation, definitive causal relationships require validation by prospective cohort or intervention studies. The relatively limited sample size, particularly in glucose subgroups, may have reduced statistical power to detect subtle effects. Exploration of immune mechanisms was confined to cell counts; future work should extend to cellular function, metabolomics, and transcriptomics to delineate the molecular mechanisms by which hyperglycemia impairs immune clearance. Additionally, the single-center design restricts generalizability, and the absence of long-term glucose indicators (e.g., HbA1c) precluded comprehensive assessment of the impact of glucose fluctuations. Future studies should expand sample sizes, systematically collect biospecimens, and integrate multi-omics data to further elucidate the pathophysiology of this comorbidity.

DM, particularly sustained hyperglycemia, is a key driver of delayed bacterial clearance and treatment failure in XDR patients. Beyond exacerbating tissue damage, it fundamentally sustains high bacterial loads and impairs pathogen clearance capacity. This study reveals a clear dose–response relationship between blood glucose levels and clinical outcomes, confirming that strict glucose intervention can effectively disrupt this pathological link in the early stages of treatment. Successful XDR treatment relies not only on effective antibacterial agents but also on host milieu homeostasis, with DM transcending its traditional comorbidity role to become a modifiable “pathophysiological regulator.” By shifting part of the therapeutic focus from irreversible bacterial drug resistance to the adjustable host metabolic state, this study clarifies the core mechanism by which DM exacerbates drug-resistant tuberculosis progression and establishes intensified glucose management as an indispensable strategy to promote bacterial clearance, improve patient outcomes, and reduce disease burden—opening a clinically viable host-directed pathway to address XDR, one of the most pressing global public health challenges.

## Data Availability

The original contributions presented in the study are included in the article/Supplementary material, further inquiries can be directed to the corresponding authors.
