# Supplementary material for: Glucose driven bacterial persistence in extensively drug-resistant tuberculosis with diabetes
Source: Front Public Health. 2026 Mar 26;14:1746879. doi: 10.3389/fpubh.2026.1746879 (PMC13062200; doi:10.3389/fpubh.2026.1746879)
Supplement: Supplementary file 1 [file Supplementary_File_1.docx]

Supplementary Material

# Supplementary Figures and Tables

## Supplementary Figures


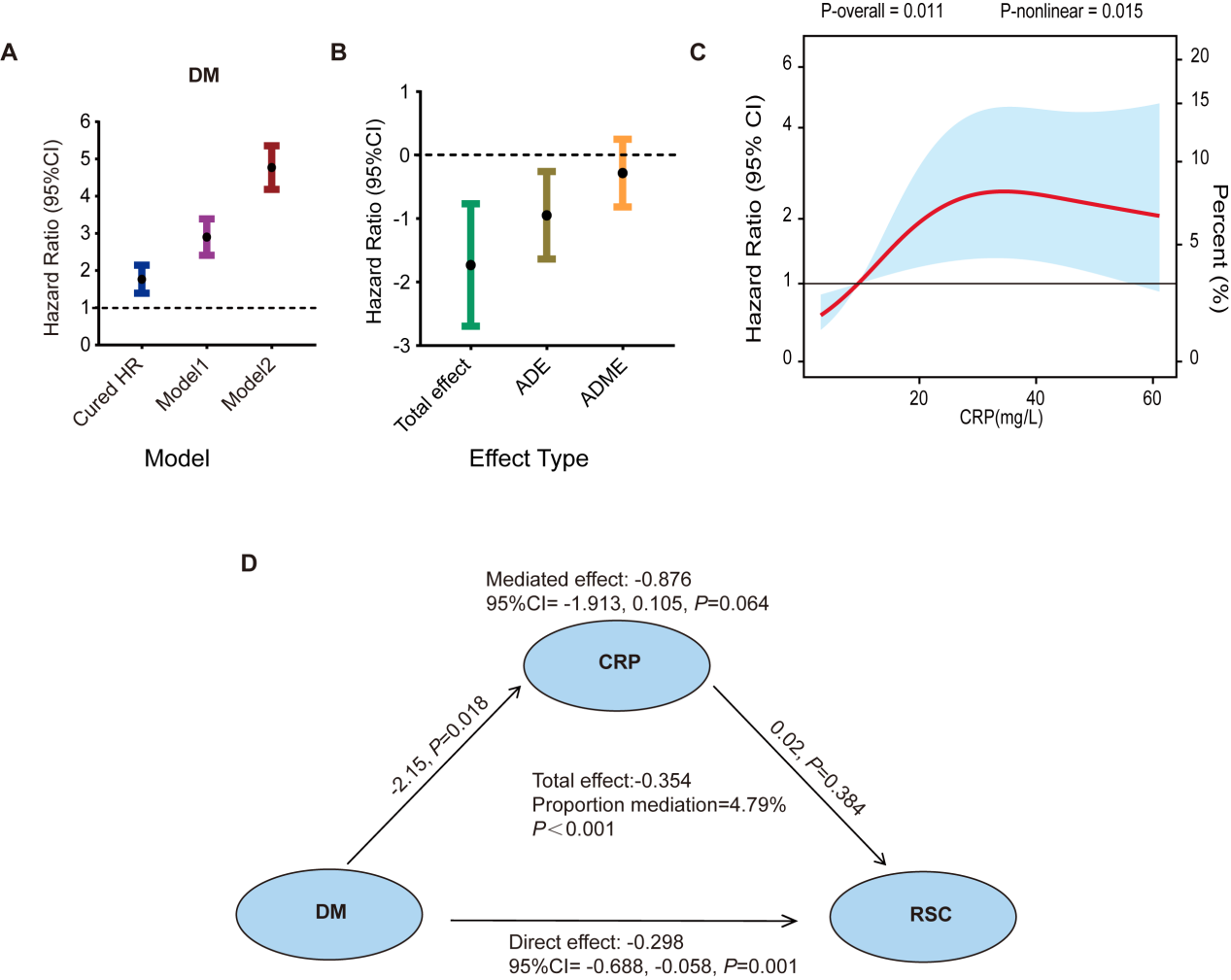


**Figure S1. Mediation analysis of C-reactive protein (CRP) on the relationship between diabetes and bacterial clearance.** (A) Progressive adjustment in Cox models reveals the persistent association between diabetes and delayed bacterial clearance after accounting for clinical confounders. (B) Mediation analysis using the Bootstrap method confirms that a significant portion of diabetes' total effect is indirectly mediated through elevated CRPlevels (ACME), alongside a residual direct effect (ADE). (C) The continuous relationship between rising CRP levels and increasing risk of delayed clearance is visualized using a restricted cubic spline model. (D) Schematic of the established mediation pathway, quantifying the significant indirect effect of diabetes via CRP, as tested by Bootstrap. All associations were significant at P < 0.05.

## **Supplementary Tables**

| **Table S1.Comparison of Non parametric Tests for Various Indicators between XDR and DM+XDR Groups.** | | | | | | |
| --- | --- | --- | --- | --- | --- | --- |
|  |  |  |  |  |  |  |
| variable |  | XDR （n=57） |  | DM+XDR （n=84） |  | P-value |
| WBC |  | 6.070(5.360,8.010) |  | 6.605(5.408,8.208) |  | 0.580 |
| NEUT |  | 4.210(3.050,5.850) |  | 4.440(3.343,5.540) |  | 0.606 |
| LYM |  | 1.340(1.080,1.715) |  | 1.370(1.038,1.638) |  | 0.998 |
| MONO |  | 0.500(0.395,0.705) |  | 0.570(0.395,0.750) |  | 0.634 |
| EO |  | 0.120(0.065,0.195) |  | 0.110(0.060,0.188) |  | 0.472 |
| BASO |  | 0.020(0.010,0.030) |  | 0.020(0.010,0.030) |  | 0.498 |
| CRP |  | 11.300(4.050,28.050) |  | 18.450(6.825,36.680) |  | 0.012 |
| LDH |  | 154.000(129.200,189.700) |  | 165.100(134.000,183.200) |  | 0.441 |
| HGB |  | 126.000(108.000,137.000) |  | 129.000,118.000,140.800) |  | 0.034 |
| RBC |  | 4.450(4.010,4.830) |  | 4.520(4.193,4.855) |  | 0.293 |
| PLT |  | 254.000(206.000,312.000) |  | 215.000(176.800,266.000) |  | 0.020 |
| HCT |  | 0.380(0.345,0.420) |  | 0.390(0.363,0.420) |  | 0.175 |
| MCV |  | 85.000(83.000,90.050) |  | 86.050(82.000,89.450) |  | 0.984 |
| ALB |  | 38.000(36.000,41.000) |  | 38.000(34.250,40.000) |  | 0.166 |
| PA |  | 181.000(144.000,227.000) |  | 167.000,127.800,229.800) |  | 0.498 |
| TRF |  | 2.010(1.615,2.420) |  | 1.765(1.413,2.290) |  | 0.028 |
| ALT |  | 13.000(9.000,22.000) |  | 14.000(9.000,20.000) |  | 0.999 |
| AST |  | 18.000(16.000,22.500) |  | 16.000(13.000,21.750) |  | 0.012 |
| TBIL |  | 8.100(6.200,15.100) |  | 10.050(6.725,13.550) |  | 0.622 |
| UA |  | 327.000(264.500,485.000) |  | 313.000(250.000,406.000) |  | 0.241 |
| CREA |  | 56.000(48.000,71.000) |  | 58.000(50.250,69.000) |  | 0.604 |
| CYSC |  | 0.670(0.565,0.905) |  | 0.810(0.680,0.990) |  | 0.001 |
| GLU |  | 4.400(4.150,4.800) |  | 8.850(6.600,13.480) |  | ＜0.001 |
| CD3 |  | 68.900(61.500,75.450) |  | 70.650(63.730,75.600) |  | 0.428 |
| CD16 |  | 9.710(5.795,13.620) |  | 10.190(6.495,19.700) |  | 0.152 |
| CD4 |  | 35.600(29.450,43.100) |  | 41.400(32.900,48.680) |  | 0.007 |
| CD8 |  | 22.700(17.350,30.350) |  | 18.250(12.850,24.500) |  | ＜0.001 |
| CD4/CD8 |  | 1.690(1.045,2.580) |  | 2.270(1.403,3.840) |  | 0.010 |
| IGG |  | 13.600(10.250,16.400) |  | 12.190(9.218,15.120) |  | 0.029 |
| C3 |  | 1.290(1.035,1.470) |  | 1.230(1.023,1.515) |  | 0.967 |
| C4 |  | 0.280(0.180,0.345) |  | 0.295(0.223,0.380) |  | 0.156 |

| **Table S2. Baseline characteristics of patients stratified by blood glucose levels (N=84).** | | | | | | | | |
| --- | --- | --- | --- | --- | --- | --- | --- | --- |
| variable | NG  (n=24) | | MG  (n=30) | | HG  (n=30) | |  | P-value |
| **Age** | 52.79±9.87 | | 55.27±11.66 | | 51.47±10.15 | |  | 0.379 |
| **Sex** |  |  |  |  |  |  |  | 1.000 |
| female | 3 | 12.50 | 2 | 6.67 | 3 | 10.00 |  |  |
| male | 21 | 87.50 | 28 | 93.33 | 27 | 90.00 |  |  |
| BMI | 22.50±3.11 | | 22.41±3.79 | | 21.24±3.11 | |  | 0.289 |
| **Sputum smear** |  |  |  |  |  |  |  | 0.912 |
| 1+ | 9 | 37.50 | 9 | 30.00 | 6 | 20.00 |  | 0.233 |
| 2+ | 4 | 16.67 | 5 | 16.67 | 9 | 30.00 |  | 0.343 |
| 3+ | 9 | 37.50 | 10 | 33.33 | 9 | 30.00 |  | 0.577 |
| 4+ | 2 | 8.33 | 6 | 20.00 | 6 | 20.00 |  | 0.277 |
| Sputum smear turns negative | 15 | 62.50 | 15 | 50.00 | 8 | 26.67 |  | 0.013 |
| Median time for sputum smear to turn negative | 6(2.25,12) | | 12(4.5,24) | | 14(4.75,28) | |  | 0.001 |
| **CT features of lungs** |  |  |  |  |  |  |  |  |
| cavity | 15 | 62.50 | 28 | 93.33 | 28 | 93.33 |  | 0.007 |
| pleural effusion | 10 | 41.67 | 14 | 46.67 | 10 | 33.33 |  | 0.570 |
| **The affected area of the lesion** |  |  |  |  |  |  |  | 1.000 |
| left/right | 4 | 16.67 | 3 | 10.00 | 5 | 16.67 |  |  |
| both | 20 | 83.33 | 27 | 90.00 | 25 | 83.33 |  |  |
| Tuberculosis treatment history | 24 | 100.00 | 30 | 100.00 | 30 | 100.00 |  | 1.000 |
| **Adverse drug reactions** | 10 | 41.67 | 13 | 43.33 | 15 | 50.00 |  | 0.855 |
| **cure** | 16 | 66.67 | 13 | 43.33 | 6 | 20.00 |  | ＜0.001 |
|  |  |  |  |  |  |  |  |  |
